# APOC1 knockdown induces apoptosis and decreases angiogenesis in diffuse large B-cell lymphoma cells through blocking the PI3K/AKT/mTOR pathway

**DOI:** 10.17305/bb.2024.11550

**Published:** 2025-01-23

**Authors:** Jing Gao, Xiaojuan Lu, Guanglei Wang, Tanling Huang, Zhongyu Tuo, Weiwei Meng

**Affiliations:** 1 Clinical Laboratory, Shenzhen Baoan Shiyan People’s Hospital, Guangdong Province, China

**Keywords:** Diffuse large B-cell lymphoma, DLBCL, apolipoprotein C1, APOC1, human umbilical vein endothelial cells, HUVECs, malignant biological behaviors, angiogenesis, PI3K/AKT/mTOR pathway

## Abstract

Diffuse large B-cell lymphoma (DLBCL) is a highly heterogeneous metastatic lymphoma that can be treated by targeting angiogenesis. Apolipoprotein C1 (APOC1) plays a significant role in the proliferation and metastasis of various malignant tumors; however, its role in DLBCL—particularly its effects on angiogenesis—remains largely unexplored. This study investigates the correlation between APOC1 expression and patient prognosis in DLBCL. Using APOC1 gene knockdown, apoptosis, migration, and invasion were assessed through flow cytometry, the EDU assay, wound healing, and Transwell assays. Additionally, human umbilical vein endothelial cells (HUVEC) angiogenesis was evaluated. Advanced techniques, such as immunofluorescence, TUNEL assay, and immunohistochemical labeling were employed to analyze the effects of APOC1 knockdown on the PI3K/AKT/mTOR signaling pathway and tumor formation in nude mice. Results showed that APOC1 is overexpressed in DLBCL tissues and cells, with high APOC1 levels associated with poor patient prognosis. *In vitro* experiments revealed that APOC1 knockdown increased apoptosis and inhibited cell proliferation, migration, invasion, HUVEC angiogenesis, and PI3K/AKT/mTOR signaling pathway protein expression in DLBCL cells. Similarly, *in vivo* studies demonstrated that APOC1 knockdown significantly reduced tumor growth, angiogenesis-related proteins, and phosphorylated PI3K/AKT/mTOR pathway proteins in nude mice. APOC1 knockdown promotes apoptosis and suppresses angiogenesis in DLBCL cells by inhibiting the PI3K/AKT/mTOR pathway.

## Introduction

Diffuse large B-cell lymphoma (DLBCL) is the most common type of non-Hodgkin lymphoma (NHL) worldwide [1]. Clinically, most patients with DLBCL present with a rapidly developing mass involving one or more lymph nodes, often accompanied by extranodal involvement. Approximately 40% of patients exhibit extranodal disease [2, 3]. DLBCL is highly heterogeneous, displaying significant variability in biological behavior, histomorphology, clinical presentation, and patient response to therapy, which results in diverse prognoses [4, 5]. While studies have shown that the combination of immunotherapy and chemotherapy with Rituximab, cyclophosphamide, doxorubicin hydrochloride, vincristine sulfate, and prednisone (R-CHOP) significantly improves clinical outcomes, 30%–40% of patients remain resistant to this regimen or experience relapse following treatment [6, 7]. Consequently, identifying biological targets or markers to better treat or evaluate DLBCL is essential for improving overall patient survival. Apolipoprotein C1 (APOC1), a member of the apolipoprotein C family, has a molecular weight of 6.6 kDa [8]. APOC1 is primarily involved in the metabolism of very-low-density and high-density lipoproteins, playing a critical role in plasma lipoprotein metabolism [9–12]. Recent investigations suggest that APOC1 is a potential therapeutic target as well as a valuable diagnostic, prognostic, and immune biomarker for various cancers. It has been shown to influence tumor proliferation and metastasis and is strongly expressed in several malignancies, where it is considered an oncogene [8, 13–15]. Based on this evidence, we hypothesized that APOC1 is closely linked to the development and progression of DLBCL. However, studies examining the specific role of APOC1 in DLBCL remain scarce. During solid tumor formation, increased oxygen consumption, nutrient depletion, and the accumulation of metabolic byproducts create a hypoxic microenvironment unfavorable for tumor cell proliferation [16]. Neovascularization, which provides oxygen and nutrients, becomes critical for tumor cell survival and is a key factor in cancer progression [17]. Consequently, inhibiting angiogenesis is an effective strategy to suppress tumor growth and proliferation. Notably, downregulating APOC1 expression has been shown to reduce angiogenesis and inhibit the spread of clear cell renal cell carcinoma [18]. However, the precise mechanisms by which APOC1 influences angiogenesis and cancer progression require further investigation. The PI3K/AKT/mTOR signaling pathway is a well-known regulator of essential cellular processes, including proliferation, survival, metabolism, motility, and autophagy [15, 16]. Dysregulation and hyperactivation of this pathway are observed in nearly all cancers, contributing to tumor progression by promoting the G1-to-S phase transition in the cell cycle, thereby accelerating cancer growth [17]. Additionally, the PI3K/AKT/mTOR pathway enhances tumor cell proliferation, invasion, metastasis, and angiogenesis while inhibiting apoptosis and increasing resistance to treatment [18–22]. Targeting the PI3K/AKT/mTOR pathway has led to the development of several therapeutic agents used in cancer treatment [23]. Importantly, studies suggest that apolipoproteins can inhibit PI3K/AKT/mTOR-mediated angiogenesis in breast cancer [24], which supports the hypothesis that APOC1 may play a role in the malignant progression and angiogenesis of DLBCL via this signaling pathway. In this study, we explored the correlation between APOC1 expression levels and DLBCL patient prognosis, highlighting its clinical value in assessing disease progression and outcomes. We also investigated the functional role of APOC1 in DLBCL development and its potential molecular mechanisms through *in vitro* and *in vivo* experiments. These findings aim to contribute to improved early screening and treatment strategies for DLBCL.

## Materials and methods

### Bioinformatics predictions

The APOC1 level in DLBCL samples from the TCGA database was analyzed using GEPIA2, accessible at http://gepia2.cancer-pku.cn/#index. Kaplan–Meier survival analysis was performed to evaluate the impact of APOC1 expression levels on the survival outcomes of DLBCL patients.

### Clinical specimens

Lymph node samples were collected from 46 patients with DLBCL45 and 13 patients with reactive hyperplasia of lymph nodes (RHL) at Shenzhen Baoan Shiyan People’s Hospital. The hospital’s Ethics Committee approved the study (SYLL017) after obtaining informed consent from all participants. The 46 DLBCL patients underwent standard staging procedures, including baseline clinical and organ function assessments (e.g., age, gender, physical performance, lactate dehydrogenase levels, and liver function) and Ann Arbor staging [22]. Diagnostic evaluations included bone marrow aspiration and biopsy, CT, and positron emission tomography-CT (PET-CT). Physical performance was assessed using the Eastern Cooperative Oncology Group (ECOG) score, while prognostic status was determined using the International Prognostic Index (IPI) [22]. Pathologic staging was classified based on the Hans algorithm. Ann Arbor staging results showed that among the 46 DLBCL patients, five were in stage I, 18 in stage II, 14 in stage III, and nine in stage IV. All DLBCL patients were followed up by phone for five years to assess prognosis. Tissue samples were fixed in 10% neutral buffered formalin for a minimum of 24 h.

### Animal specimens

Twelve BALB/c male nude mice (4–6 weeks old) were purchased from the Shanghai Laboratory Animal Research Center, China. The mice were housed in a controlled environment at 25 ^∘^C with 50% relative humidity and a 12-h light/dark cycle. They had ad libitum access to food and water. All animal experiments were approved by the Ethics Committee of Shenzhen Bao’an Shiyan People’s Hospital (Approval No. SYLL103) and conducted in accordance with guidelines for animal welfare and the ethical use of animals in cancer research.

### Cell lines and cultures

Normal human B-lymphocyte cell line GM2878, DLBCL cell lines (SU-DHL-4 and SU-DHL-8), and human umbilical vein endothelial cells [HUVECs] were obtained from the Chinese Academy of Sciences’ cell bank (Shanghai, China). GM2878 and DLBCL cell lines were cultured in RPMI-1640 medium (ORCPM0110B, ORiCells Biotechnology, Shanghai, China) supplemented with 10% fetal bovine serum. The SU-DHL-4 and SU-DHL-8 cells were switched to serum-free DMEM and cultivated for 48 h. Tumor-conditioned media (SU-DHL-4 CM and SU-DHL-8 CM) was collected, centrifuged, and filtered before use. HUVECs were subsequently cultured in SU-DHL-4 CM or SU-DHL-8 CM [20]. All cells were maintained in a humidified incubator at 37 ^∘^C with 5% CO_2_. Cells were passaged once adherent growth density exceeded 80%.

### Cell transfection

Ribobio (Guangzhou, China) constructed knockdown APOC1 (sh-APOC1) and negative control (sh-NC) vectors. These vectors were subsequently transfected into DLBCL cell lines (SU-DHL-4 and SU-DHL-8) using Lipofectamine 3000 (L3000001, Invitrogen, Austin, TX, USA), following the manufacturer’s instructions. After transfection, the cells were incubated for 48 h before being subjected to further experiments.

### RT-qPCR test

A Trizol kit (DP424, TIANGEN, Beijing, China) was used to isolate total RNA from cells and tissues. cDNA synthesis was performed using the PrimeScript RT Reverse Transcription Kit (RR047 A, Takara, Tokyo, Japan). The expression of APOC1 and the endogenous control β-actin was analyzed using an ABI PRISM 7300 RT-PCR system (7300, ABI, Carlsbad, CA, USA) with SYBR Green dye (4309155, Applied Biosystems, DE, USA) following the manufacturer’s protocol. Gene expression levels were quantified using the 2^−ΔΔCt^ method with the following primers:

APOC1: F: 5′-TCAACGTGCTTTGGTCCATCT-3′; R: 5′-GAAAACCACTCCCTGTGGGG-3′.

β-actin: F: 5′-ATCACTATTGGCAACGAGCG-3′; R: 5′-ACTCATCGTACTCCTGCTTG-3′.

### Western blot analysis

Total proteins from tissues and cells were extracted using RIPA buffer (Yeasen, Shanghai, China). Protein concentrations were determined prior to heat denaturation. Proteins were then separated via electrophoresis and transferred onto PVDF membranes. The membranes were blocked for 15 min in Pierce protein-free blocking buffer (37573, Thermo Fisher, Waltham, MA, USA) to prevent nonspecific binding. Next, the membranes were incubated overnight at 4 ^∘^C with the following primary antibodies: anti-APOC1 (1:1000, ab189866, Abcam), anti-Cleaved caspase-3 (1:500, ab32042, Abcam), anti-Cleaved caspase-9 (1:1000, #9505, CST), anti-MMP-2 (1:1000, ab97779, Abcam), anti-MMP-9 (1:1000, ab76003, Abcam), anti-VEGFA (1:1000, ab46154, Abcam), anti-PI3K (1:1000, ab302958, Abcam), anti-p-PI3K (1:1000, #13857, phospho Ser249, CST), anti-Akt (1:1000, ab8805, Abcam), anti-p-Akt (1:1000, ab38449, phospho T308, Abcam), anti-mTOR (1:10,000, ab134903, Abcam), anti-p-mTOR (1:1000, ab109268, phospho S2448, Abcam), and anti-β-actin (1:1000, ab8227, Abcam). After three washes with TBST, the membranes were incubated for 1 h at room temperature with an HRP-labeled goat anti-rabbit IgG secondary antibody. Target proteins were detected using ECL and visualized on a chemiluminescent imaging system (Tanon, Shanghai, China). Relative protein quantification was performed as part of the analysis.

### GFP fluorescence detection

Green fluorescent protein (GFP) is an autofluorescent protein that can be stably expressed in various cell types. It is widely used in applications, such as gene expression analysis, protein localization studies, cell tracking, and labeling. When exposed to UV or blue light, GFP’s endogenous fluorescent moiety emits a stable and easily visible green fluorescence. To evaluate transfection efficiency, we placed the transfected DLBCL cells in dark areas and monitored GFP expression using a MICA fluorescent microscope.

### Flow cytometry

Cells were collected and analyzed for apoptosis using the Annexin V-FITC/PI Apoptosis Detection Kit (40302ES20, Yeasen), following the manufacturer’s instructions. After treatment under different conditions, the cells were washed with PBS and resuspended in 100 µL of binding buffer. They were then incubated with Annexin V-FITC and PI for 10 min at 25 ^∘^C, protected from light. Following staining, 400 µL of binding buffer was added to each sample, which was subsequently analyzed within 1 h using a BD FACSCelesta™ flow cytometer (BD, NJ, USA).

### Adenosine triphosphate (ATP) assay

The ATP concentration in DLBCL cells was measured using an ATP detection kit (S0026, Beyotime, Shanghai, China). Following the manufacturer’s instructions, the cell supernatant was centrifuged, and the substrate solution was added. The chemiluminescence intensity of the solution was then measured with a chemiluminescence analyzer (2805880, Thermo Fisher). The ATP concentration was subsequently calculated based on a standard curve.

### Mitochondrial membrane potential detection

According to the instruction manual for the C2006 kit (Beyotime), membrane potential alterations in DLBCL cells were detected using the JC-1 probe. When the membrane potential is high, JC-1 aggregates in the mitochondrial matrix, forming J-aggregates that emit red fluorescence. Conversely, when the membrane potential is low, JC-1 remains in its monomeric form, emitting green fluorescence. To perform the assay: Aspirate the culture medium from the six-well plate and wash the cells with PBS; prepare the working solution by combining the JC-1 reagent with the cell culture medium, mix thoroughly, and add it to the cells. Incubate the cells at 37 ^∘^C for 20 min. After incubation, aspirate the supernatant, wash the cells twice with JC-1 buffer, and then add 2 mL of fresh cell culture medium. Examine the cells under a fluorescence microscope to assess the fluorescence signals.

### CCK-8 test

Transfected DLBCL cells and conditioned medium-treated HUVECs were digested with trypsin, centrifuged, and counted before being seeded into 96-well plates at a density of 1 × 10^ImEquation2^ cells/well. A total of 100 µL of culture medium was added to each well, and the plates were incubated at 37 ^∘^C with 5% CO_2_ for 0, 24, 48, and 72 h. After the incubation periods, 10 µL of CCK-8 solution (HY-K0301, MedChem Express, Monmouth Junction, NJ, USA) was added to each well and mixed thoroughly. The plates were then incubated for an additional 2 h. Finally, the absorbance (OD value) at 450 nm was measured for each well using a microplate reader.

### EDU test

RiboBio’s EDU kit (C10310-1) was used to detect cell growth. Cell suspensions were seeded into 24-well plates at a density of 2 × 10^ImEquation4^ cells per well and cultured for 24 h under various treatment conditions. Afterward, the culture media were removed, and the cells were rinsed with PBS. They were incubated with a 10 µM EDU staining solution for 1 h at room temperature, protected from light. The cells were then washed with PBS, fixed with 4% paraformaldehyde for 15 min, and permeabilized with 0.3% Triton X-100 in PBS. Next, the cells were incubated with a Click reaction solution for 30 min at room temperature, away from light. Finally, the nuclei were stained with DAPI for 10 min in the dark, followed by fluorescence observation and imaging.

### Transwell assay

To investigate cell invasion, Transwell chambers were pre-coated with 50 µL of Matrigel Matrix Gel (354234, Corning Inc., Corning, NY, USA) and left at room temperature for 30 min to allow polymerization. The chambers were then placed in 24-well plates, with each chamber receiving a serum-free cell suspension. RPMI-1640 medium containing 15% FBS was added to the lower compartment of the wells. The plates were incubated at 37 ^∘^C in a 5% CO_2_ atmosphere for 24 h. Cells that migrated to the lower surface of the membrane were fixed in 4% paraformaldehyde for 30 min and stained with 0.1% crystal violet (C0121, Beyotime) for another 30 min. The number of migrating or invading cells was quantified using an inverted microscope (DMi8 S, Leica, Wetzlar, Germany).

### Wound healing assay

HUVECs cultured with conditioned media were digested with trypsin, resuspended in PBS, and seeded into 6-well plates at a density of 1 × 10^6^ cells per well. Once the cells adhered to the plate surface and reached confluence, a 200 µL sterile pipette tip was used to draw a uniform straight line perpendicular to the bottom of the plate. Detached cells were washed away with PBS, and the remaining cells were cultivated in serum-free medium for 24 h. Photographs were taken using microscopy at 0 and 24 h, and the scratch widths at both time points were measured using ImageJ software to calculate the cell migration rate for each group.

### Angiogenesis assay

To prepare for the experiment, 300 µL of matrix gel (C0383, Beyotime) was added to pre-cooled 48-well plates and allowed to cure at 37 ^∘^C for 30 min. HUVECs (1 × 10^ImEquation7^ cells/well) were then seeded onto the matrix gel in 200 µL of either SU-DHL-4 CM or SU-DHL-8 CM. The plates were incubated at 37 ^∘^C for 8 h. Angiogenesis was analyzed using an inverted microscope, and the tube-forming ability of HUVECs was quantified by counting the total number of branch points formed.

### Immunofluorescence assay

SU-DHL-4 and SU-DHL-8 cells were fixed with 4% paraformaldehyde and 0.5% Triton X-100 (P0096, Beyotime) and then blocked with 1% fetal bovine serum. The cells were incubated overnight at 4 ^∘^C with the following primary antibodies: rabbit anti-APOC1 (1:1000, ab189866, Abcam), anti-p-PI3K (1:1000, #17366, CST), anti-p-Akt (1:1000, ab38449, Abcam), and anti-p-mTOR (1:1000, ab109268, Abcam). Afterward, they were treated with Alexa Fluor^®^ 488-labeled goat anti-rabbit IgG (1:1000, ab150077, Abcam) at 37 ^∘^C for 2 h. Finally, the cell nuclei were stained with DAPI, and fluorescent confocal microscopy was used to localize APOC1 and assess the expression levels of PI3K/AKT/mTOR pathway proteins.

### Experiments with tumors in living animals

SU-DHL-4 cell suspensions were prepared and adjusted to a concentration of 5 × 10^7^ cells/mL. Nude mice were randomly divided into two groups (sh-NC and sh-APOC1), with each group containing six mice. Each mouse received a subcutaneous injection of 100 µL of tumor cell solution on the right flank and was maintained in a pathogen-free (SPF) environment. Tumor volumes were measured every seven days. On the 28th day, the mice were euthanized, and the tumors were excised, photographed, and weighed to assess tumor mass.

### TUNEL assay

Apoptosis in tumor tissues was measured using the TUNEL kit (C1091, Beyotime) following the manufacturer’s instructions. Tumors from mice were fixed in 4% paraformaldehyde, dried, embedded in paraffin, and sectioned into 4 µm slices. Tissue sections were deparaffinized with xylene, dehydrated using an ethanol gradient, and rinsed with PBS before each subsequent step. To prepare the tissues, sections were incubated with 20 µg/mL DNase-free proteinase K solution at room temperature for 30 min to allow reagent penetration into the nucleus. Endogenous peroxidase activity was quenched by soaking the tissues in 3% H_2_O_2_ for 15 min. Afterward, sections were stained with 50 µL of TUNEL reaction mix and incubated at 37 ^∘^C for 60 min, protected from light. DAPI staining solution (C1005, Beyotime) was applied for 5 min at room temperature, also protected from light. Finally, slices were treated with an anti-fluorescence quenching agent and analyzed for TUNEL positivity.

### Immunohistochemistry

Tissue sections were dehydrated using an ethanol gradient, subjected to antigen retrieval with citrate (C1032, Solarbio), and blocked at room temperature with avidin/biotin blocking buffer (C-0005, HaoRan Biotech, Shanghai, China). The sections were then incubated overnight at 4 ^∘^C with primary antibodies, including Ki67 (1:200, ab232784, Abcam), CD31 (1:2000, ab182981, Abcam), and VEGFA (1:100, ab52917, Abcam). After washing, the appropriate secondary antibody (1:500, ab150077, Abcam) was applied and incubated at room temperature for 1 h. The sections were subsequently stained with a streptavidin–horseradish peroxidase complex and hematoxylin (C0107, Beyotime). Finally, they were dehydrated again using an ethanol gradient, permeabilized with xylene, and sealed with neutral gum. Microscopic examination and imaging were then performed.

### Ethical statement

The ShenZhen Baoan Shiyan People’s Hospital Ethics Committee approved above animal trials, as well as they followed criteria for animal welfare and use in cancer research.

### Statistical analysis

The results of our trials were analyzed using SPSS 26.0 (SPSS Inc., Chicago, IL, USA). Data are presented as mean ± standard error. Group comparisons were conducted using ANOVA, *t*-tests, and chi-squared tests. All experiments yielded statistically significant results (*P* < 0.05).

## Results

### APOC1 is abundantly expressed in DLBCL tissues and affects tumor prognosis

**Figure 1.**
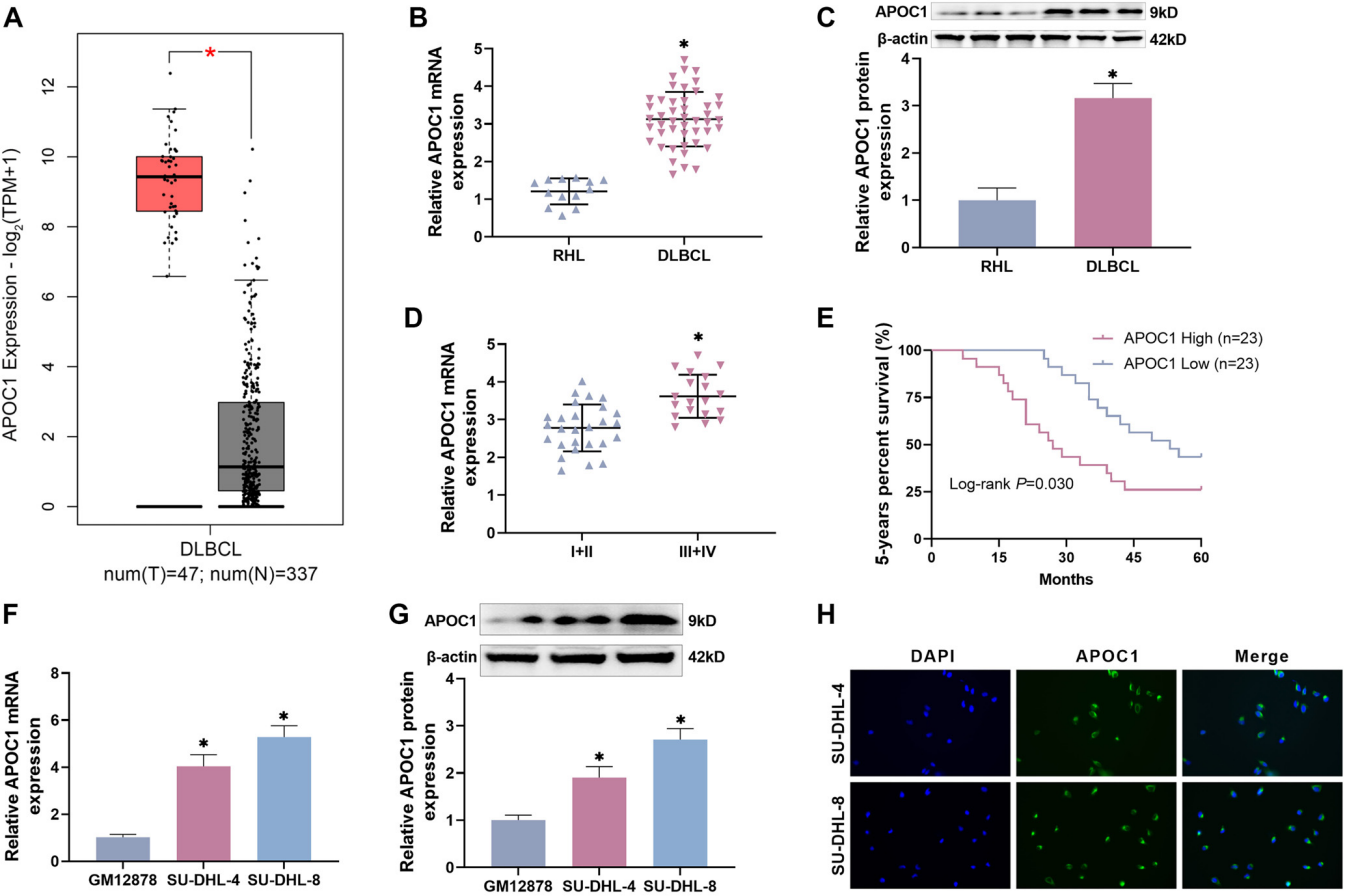
**APOC1 is abundantly expressed in DLBCL tissues and linked to tumor growth.** (A) TCGA online analysis revealed a high expression of APOC1 in the DLBCL database; (B) APOC1 mRNA expression was substantially higher in DLBCL tissues than in RHL samples, as determined by RT-qPCR; (C) Western blot analysis revealed that APOC1 protein expression was substantially higher in DLBCL tissues than in RHL tissues; (D) Stage III+IV DLBCL samples showed considerably greater levels of APOC1 expression than stage I+II DLBCL samples; (E) High APOC1 expression level is connected with a bad prognosis in DLBCL patients; (F and G) DLBCL cell lines had considerably greater APOC1 expression levels than GM2878 cells; (H) Immunofluorescence demonstrated that APOC1 is found in the cytoplasm. APOC1: Apolipoprotein C1; DBLCL: Diffuse large B-cell lymphoma; RHL: Reactive hyperplasia of lymph nodes.

To evaluate the clinical significance of APOC1 in DLBCL, we first analyzed APOC1 expression using TCGA data, which revealed significantly higher levels of APOC1 in DLBCL patients compared to normal human samples (Figure 1A). Next, we assessed APOC1 expression in lymphoma tissues from 46 DLBCL patients using RT-qPCR and Western blot, with lymph node tissues from 13 RHL patients (a benign tumor) serving as negative controls. The results (Figure 1B and 1C) showed that APOC1 expression was markedly higher in DLBCL samples than in RHL samples. To explore the relationship between APOC1 expression and DLBCL symptoms, we analyzed baseline clinical data, classifying patients into stages I–IV based on Ann Arbor staging. APOC1 levels were significantly higher in stage III+IV patients compared to those in stages I+II (Figure 1D). Kaplan–Meier survival analysis indicated that patients with high APOC1 expression had worse prognoses than those with low expression, with a five-year overall survival rate below 25% (Figure 1E). These findings suggest that APOC1 may play a key role in the poor outcomes of DLBCL patients. Finally, we examined APOC1 expression in DLBCL cell lines. As shown in Figure 1F and 1G, APOC1 expression was higher in DLBCL cell lines (SU-DHL-4 and SU-DHL-8) compared to normal human B lymphocytes (GM2878), consistent with the tumor tissue data. Immunofluorescence analysis further confirmed cytoplasmic localization of APOC1, as indicated by green fluorescence from Alexa Fluor^®^ 488-labeled APOC1 (Figure 1H).

### Knocking down APOC1 promotes apoptosis in DLBCL cells

**Figure 2.**
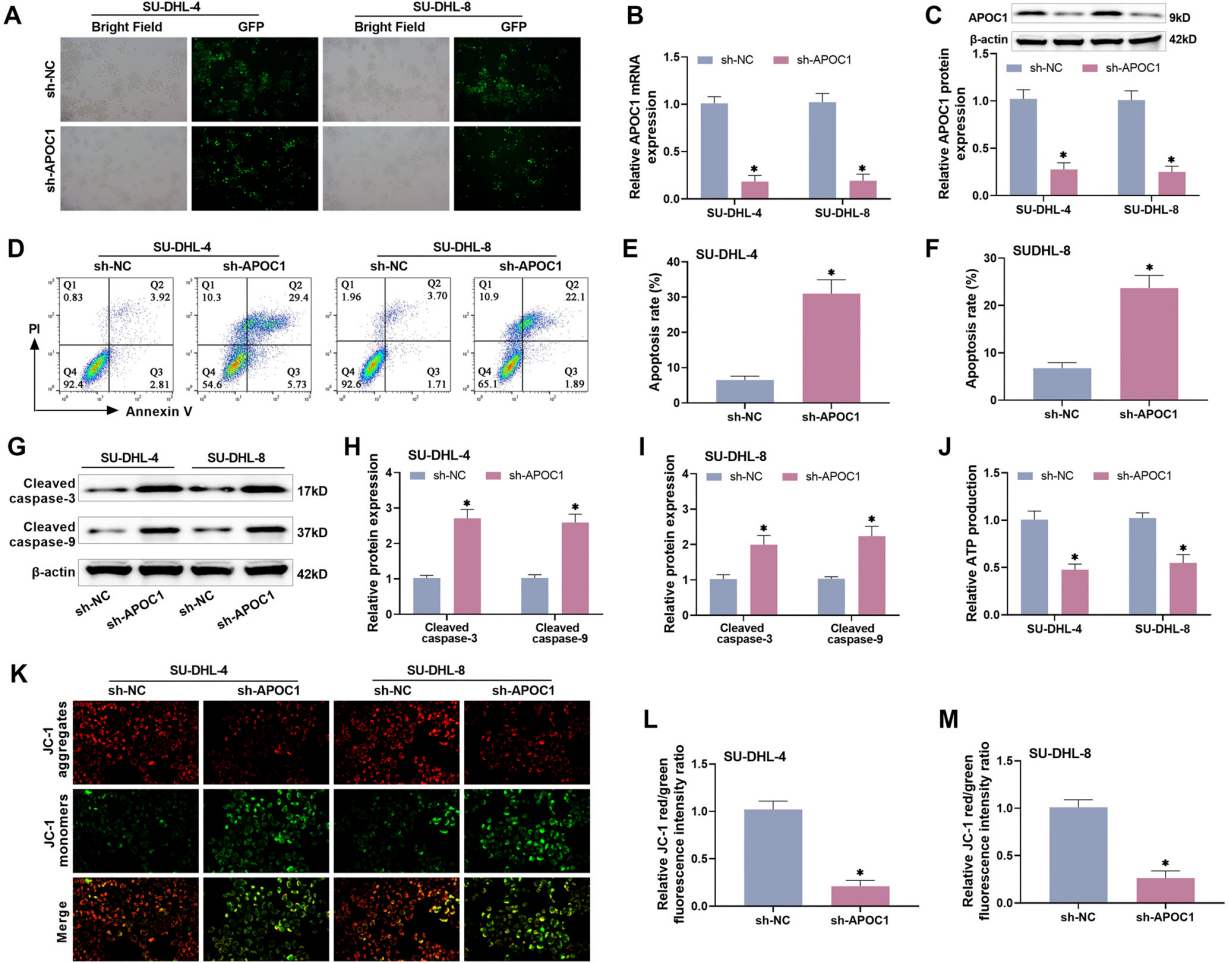
**Knocking down APOC1 promotes apoptosis in DLBCL cells.** (A) In all cases, fluorescence microscopy revealed that the expression of GFP inside the cells exceeded 80%; (B and C) RT-qPCR and Western blot analysis revealed that transfection with sh-APOC1 effectively reduced APOC1 expression levels in DLBCL cells; (D–F) Flow assay results demonstrated that knocking down APOC1 boosted the apoptotic rate of DLBCL cells; (G–I) Knocking down APOC1 led to a large rise in apoptosis-related proteins in DLBCL cells; (J) Kit for detecting ATP production; (K–M) APOC1 knockdown resulted in lower mitochondrial membrane potential and increased mitochondrial damage, as determined by the JC-1 assay. GFP: Green fluorescent protein: APOC1: Apolipoprotein C1; DBLCL: Diffuse large B-cell lymphoma; ATP: Adenosine triphosphate.

As previously stated, APOC1 expression levels may be closely associated to the development of DLBCL. To better understand its mechanism of action, we transfected sh-APOC1 and sh-NC into SU-DHL-4 and SU-DHL-8 cells, respectively, and measured APOC1’s influence on DLBCL cell apoptosis. The GFP fluorescence positive expression of cells following sh-APOC1 transfection was greater than 80% under fluorescence microscopy, indicating strong transfection efficiency, as illustrated in Figure 2A. RT-qPCR and Western blot were used to confirm that APOC1 mRNA transcription and protein expression were efficiently knocked down, respectively (Figure 2B and 2C). We employed the Annexin V/PI double labeling method to determine apoptosis by flow assay, and the findings showed that following APOC1 knockdown, the apoptosis rate of SU-DHL-4 and SU-DHL-8 cells was higher than in the sh-NC group (*P* < 0.0005, Figure 2D–2F). In addition, the apoptosis-related proteins Cleaved caspase-9 and Cleaved caspase-3 were considerably increased in the cells (*P* < 0.001, Figure 2G–2I). The content of ATP is proportional to cell viability, and its generation measures intracellular toxicity and apoptosis [21]. This work found that knocking down APOC1 reduced ATP generation in DLBCL cells, indicating a decrease in cellular ATP synthesis, severe intracellular toxicity, and increased apoptosis (Figure 2J). Another endogenous apoptotic pathway centered on mitochondria is one of the main pathways of apoptosis in tumor cells, and altered the mitochondrial membrane potential, and the large amount of Ca^2+^ entering the cell activates the endogenous mitochondrial apoptotic process [19]. In the present study, we discovered by JC-1 assay that knockdown of APOC1 resulted in a decrease of multimers (red) and an increase of monomers (green) in the mitochondria of DLBCL cells (Figure 2K–2M), demonstrating a decrease in the mitochondrial membrane potential and activation of the endogenous mitochondrial apoptotic pathway, which may be one of the causes of apoptotic cell death.

### APOC1 knockdown decreases malignant biological activities of DLBCL cells

**Figure 3.**
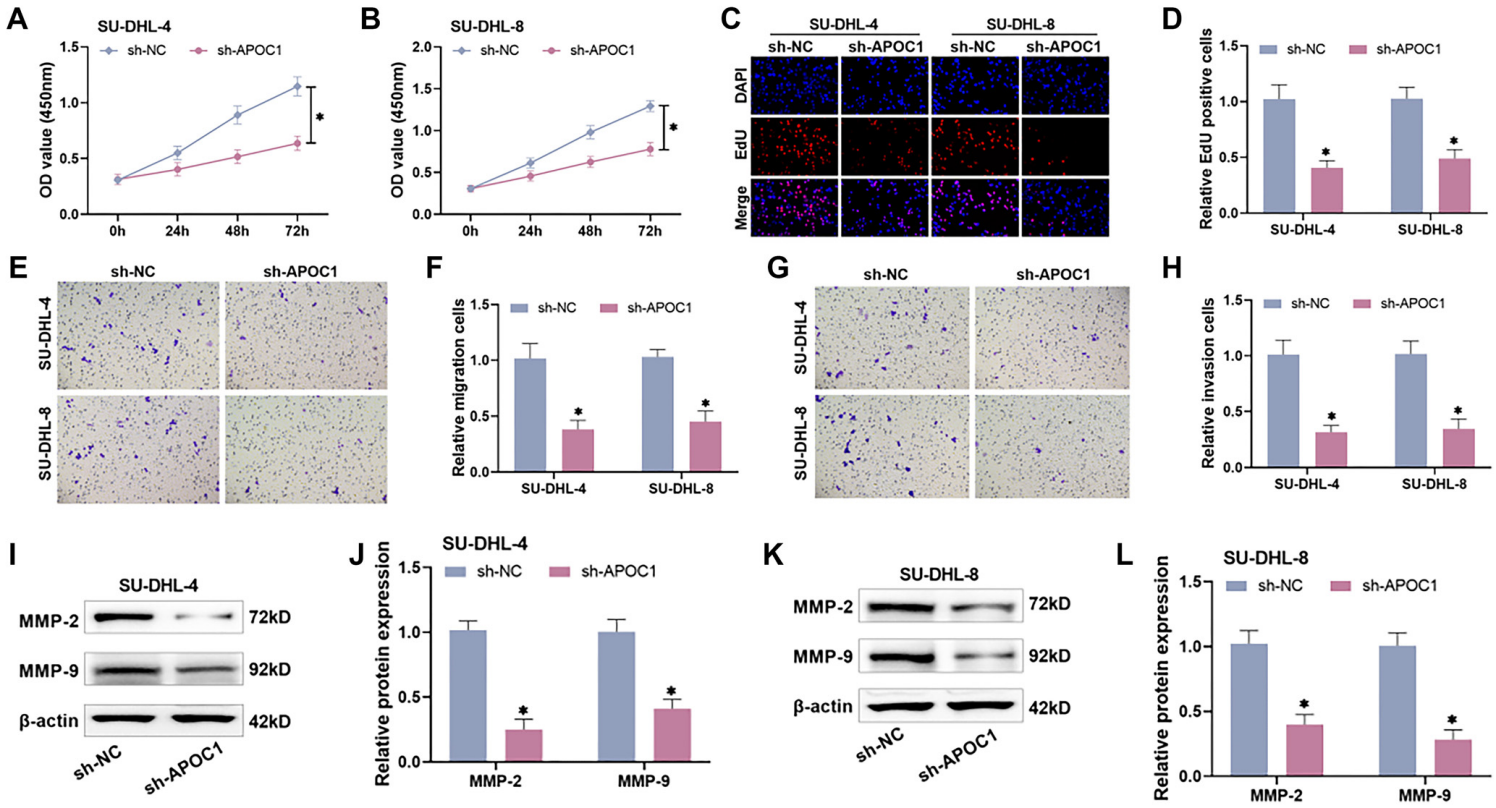
**Knockdown of APOC1 decreases DLBCL cellular carcinogenic activities.** (A and B) The CCK-8 assay demonstrated a significant decrease in proliferative vitality of DLBCL cells after suppression of APOC1; (C and D) The EdU assay demonstrated a significant decrease in the number of EdU-positive cells after suppression of APOC1; (E and F) A Transwell assay revealed that knocking down APOC1 resulted in decreased migration of DLBCL cells; (G and H) Transwell test findings demonstrated that knocking down APOC1 reduced the amount of DLBCL cell invasions; (I–L) Western blot analysis revealed that APOC1 suppression reduced the expression levels of migration and invasion proteins. APOC1: Apolipoprotein C1; DBLCL: Diffuse large B-cell lymphoma.

We also investigated the impact of APOC1 on the malignant phenotypes of DLBCL cells, including proliferation, migration, and invasion. CCK-8 assays showed that knocking down APOC1 expression in SU-DHL-4 and SU-DHL-8 cells significantly reduced their proliferative activity at 24, 48, and 72 h compared to the control group, with the most pronounced differences observed after 72 h (Figure 3A and 3B). Similarly, EdU assays revealed a marked decrease in EdU-positive DLBCL cells in the APOC1 knockdown group, confirming a reduction in cell proliferation (Figure 3C and 3D).

Transwell migration and invasion assays demonstrated that SU-DHL-4 and SU-DHL-8 cells transfected with sh-APOC1 exhibited significantly fewer migrating and invading cells compared to the control group, indicating impaired migration and invasion capabilities (*P* < 0.0002, Figure 3E–3H). MMP-2 and MMP-9, known to promote tumor invasion and metastasis by degrading the extracellular matrix and basement membrane [23], were significantly downregulated in APOC1-knockdown cells, as shown by Western blot analysis (*P* < 0.0001, Figure 3I–3L). These results highlight APOC1 as a pro-oncogenic factor in DLBCL cells. APOC1 knockdown reduced DLBCL cell proliferation, migration, and invasion while enhancing apoptosis.

### Knocking down APOC1 reduces angiogenesis in HUVECs

**Figure 4.**
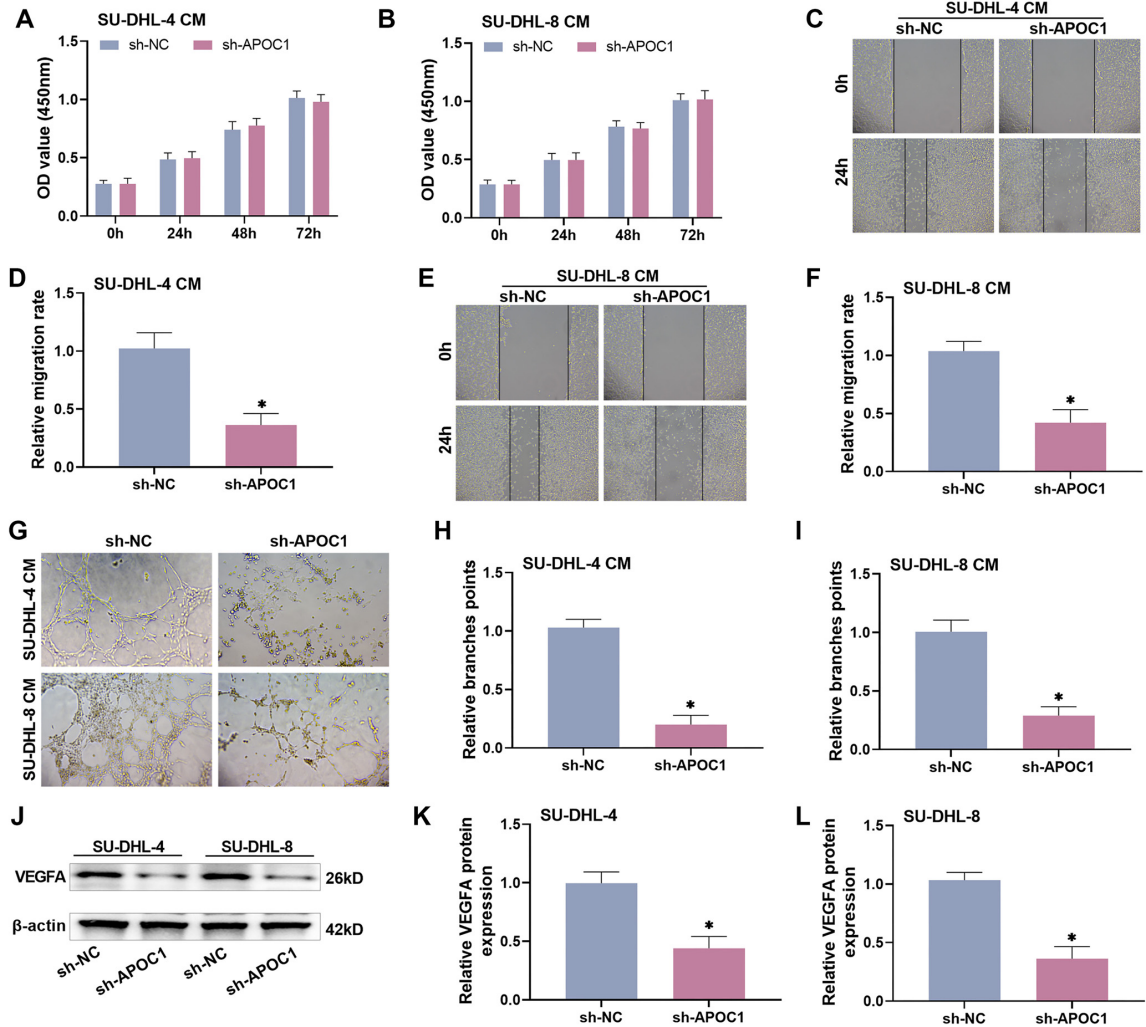
**Knockdown of APOC1 reduces angiogenesis in HUVECs.** (A–B) There was no significant change in the proliferative viability of HUVECs after treatment with conditioned media from DLBCL cells knocked down APOC1; (C–F) The wound healing experiment demonstrated that knocking down APOC1 affected the migratory ability of HUVECs; (G–I) The angiogenesis experiment demonstrated that APOC1 knockdown decreased neovascularization; (J–L) Western blot analysis revealed that APOC1 knockdown decreased VEGFA protein expression. APOC1: Apolipoprotein C1; DBLCL: Diffuse large B-cell lymphoma; HUVEC: Human umbilical vein endothelial cell.

Abnormal angiogenesis is a fundamental component of tumor formation, as neovascularization creates a hypoxic environment that promotes tumor cell invasion and hinders immune cell-mediated killing [24, 25]. To investigate whether APOC1 plays a role in angiogenesis, we examined its effects on HUVECs, given that their proliferation and migration are key steps in angiogenesis. We treated HUVECs with CM from SU-DHL-4 and SU-DHL-8 cells (SU-DHL-4 CM and SU-DHL-8 CM, respectively) after knockdown of APOC1, aiming to explore the effect of APOC1 on angiogenesis. The results showed that APOC1 knockdown did not significantly affect HUVEC proliferation or survival compared to the control group (*P* > 0.05, Figure 4A and 4B). However, the migration and invasion abilities of HUVECs were dramatically reduced following APOC1 knockdown (*P* < 0.005, Figure 4C–4F). In the angiogenesis assay, the number of tubular branch points in the sh-APOC1 group was significantly lower than in the sh-NC group, indicating that APOC1 knockdown reduced angiogenesis in HUVECs (*P* < 0.0002, Figure 4G–4I). Furthermore, Western blot analysis demonstrated that APOC1 suppression significantly reduced the levels of VEGF in HUVECs (*P* < 0.005, Figure 4J–4L). Collectively, these experimental results suggest that APOC1 knockdown decreases the angiogenic potential of HUVECs.

### Knocking down APOC1 decreases DLBCL via blocking the PI3K/AKT/mTOR pathway

**Figure 5.**
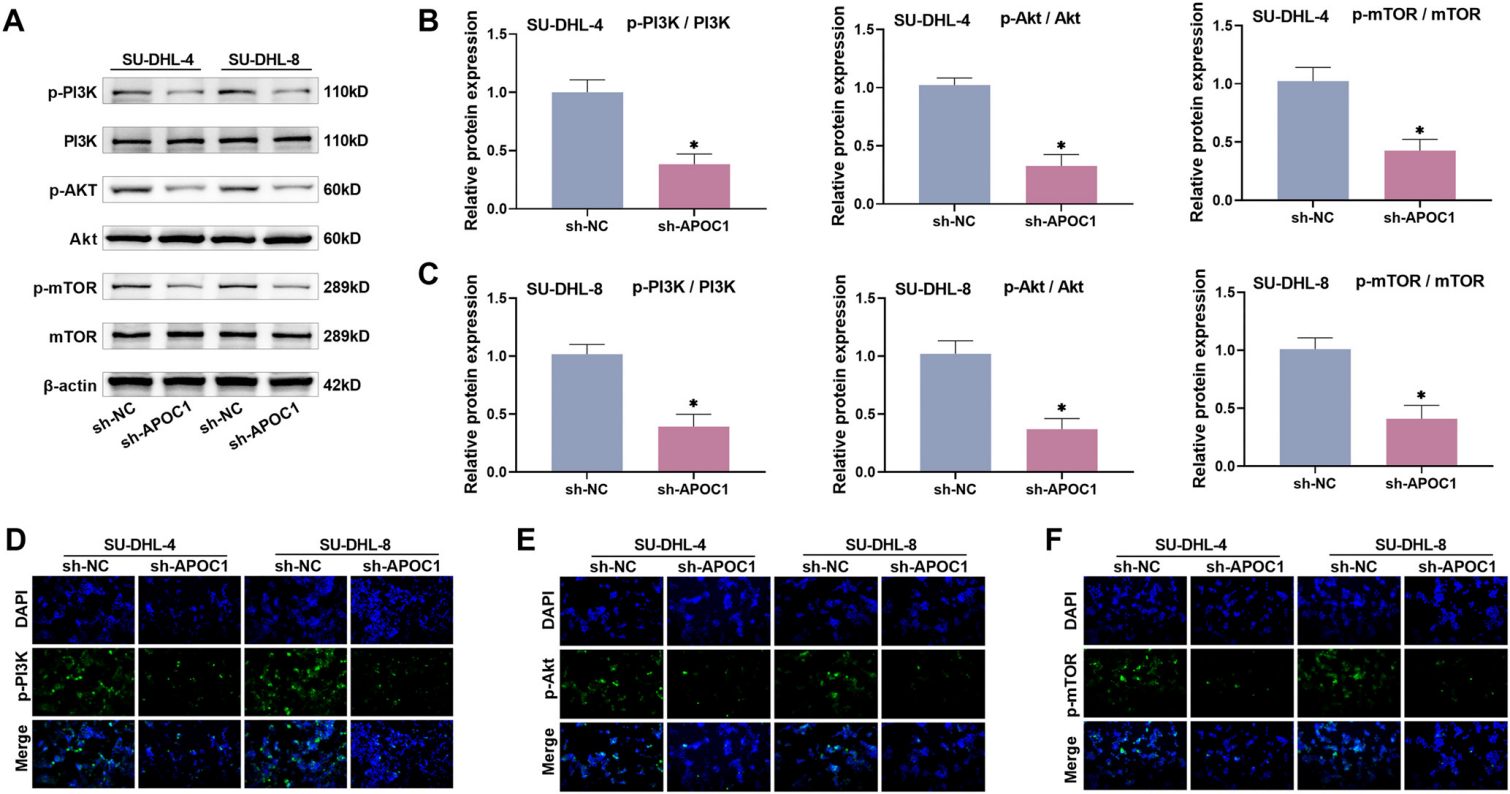
**Knockdown of APOC1 decreases DLBCL via inhibiting the PI3K/AKT/mTOR pathway.** (A–C) Western blot analysis revealed that APOC1 knockdown reduced the expression of PI3K/AKT/mTOR pathway proteins; (D–F) An immunofluorescence test demonstrated that APOC1 knockdown decreased the fluorescence expression of p-PI3K, p-AKT, and p-mTOR. APOC1: Apolipoprotein C1; DBLCL: Diffuse large B-cell lymphoma.

The PI3K/AKT/mTOR signaling pathway is a well-established regulator of numerous biological processes, including cell proliferation, differentiation, invasion, and tumor growth [26]. To investigate the role of APOC1 in modulating the PI3K/AKT/mTOR pathway in DLBCL cells, we performed Western blot analyses to assess the expression levels of pathway-related proteins, as shown in Figure 5A–5C. In DLBCL cells with APOC1 knockdown, the phosphorylation levels of key pathway proteins—phospho-Ser249-PI3K/PI3K, phospho-T308-Akt/Akt, and phospho-S2448-mTOR/mTOR—were significantly reduced compared to the sh-NC group. Furthermore, immunofluorescence staining of phosphorylated proteins (p-PI3K, p-Akt, and p-mTOR) revealed that the fluorescence intensity in the sh-APOC1 group was notably weaker than in the sh-NC group (Figure 5D–5F). These findings indicate that APOC1 knockdown inhibits the activation of the PI3K/AKT/mTOR signaling pathway.

### Knocking down APOC1 reduces tumor development and angiogenesis in nude mice *in vivo*

**Figure 6.**
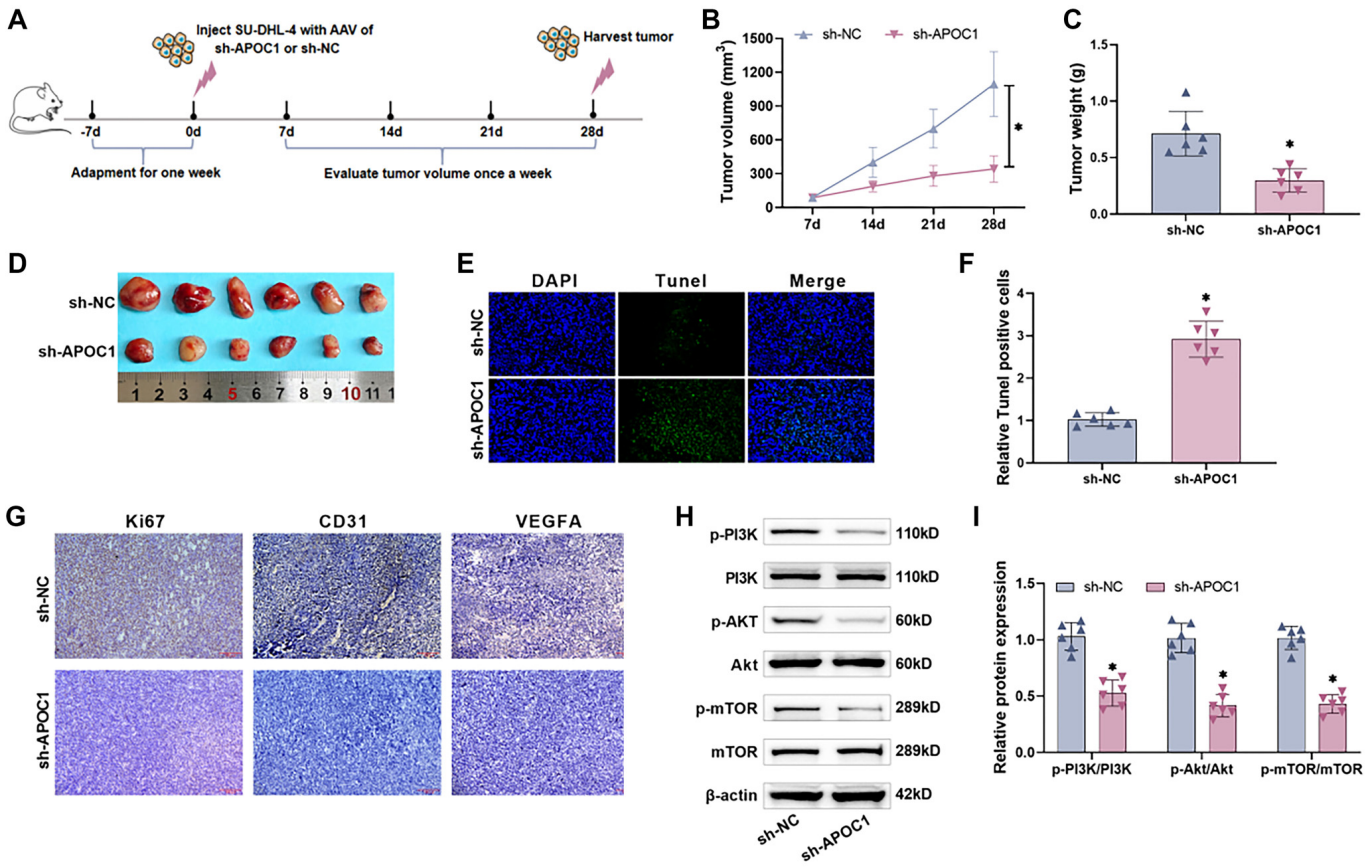
**Knocking down APOC1 reduces tumor development and angiogenesis *in vivo***. (A) Time line for nude mice culture. nude mice were acclimatized for one week prior to cell injection, and tumor volume was monitored every seven days thereafter until the nude mice were killed on the 28th day and the tumors were isolated *in vivo*; (B) Changes *in vivo* tumor volume in nude mice over the incubation period. APOC1 knockdown decreased tumor growth; (C) The tumor mass in the knockdown APOC1 group was considerably smaller than that of the control group; (D) Comparison of tumors peeled off on the 28th day. Tumor diameters in the sh-APOC1 group were considerably less than those in the sh-NC group; (E and F) The TUNEL assay demonstrated that the number of TUNEL-positive cells in the sh-APOC1 group was considerably higher than that in the sh-NC group, indicating that apoptosis of tumor cells was increased; (G) Immunohistochemistry revealed that the expression levels of Ki67, CD31, and VEGFA in tumor tissues were dramatically reduced following APOC1 knockdown, indicating that tumor cell proliferation, differentiation, and angiogenesis were blocked; (H and I) Western blot analysis revealed that APOC1 knockdown dramatically lowered the levels of p-PI3K, p-AKT, and p-mTOR proteins in tumor tissues, inhibiting the PI3K/AKT/mTOR pathway. APOC1: Apolipoprotein C1.

To investigate the effect of APOC1 protein on DLBCL tumorigenesis *in vivo*, stably transfected cells (sh-APOC1) were subcutaneously injected into BALB/c male nude mice at the junction of the dorsal neck and rear. Once tumorigenesis was visible, tumor volume was measured weekly, and tumors were collected, weighed, and photographed 28 days post-injection. Figure 6A outlines the experimental timeline. Tumors in both groups grew progressively over time. However, compared to the sh-NC group, the sh-APOC1 group exhibited slower tumor growth, with significantly reduced tumor volume and mass on day 28 (*P* < 0.001, Figure 6B and 6C). Figure 6D shows tumors isolated from the two groups. Tunel assay results revealed a significantly higher number of Tunel-positive cells in the sh-APOC1 group (*P* < 0.0001, Figure 6E and 6F), indicating an increased apoptotic rate following APOC1 knockdown. Immunohistochemistry showed weaker Ki67, CD31, and VEGFA staining in the sh-APOC1 group compared to the sh-NC group (Figure 6G), suggesting that APOC1 knockdown inhibited tumor cell proliferation and angiogenesis. Western blot analysis revealed that phosphorylated PI3K/AKT/mTOR pathway proteins were significantly reduced in the sh-APOC1 group (Figure 6H and 6I), confirming pathway inhibition. These findings demonstrate that APOC1 knockdown suppresses DLBCL tumor growth and angiogenesis *in vivo* by inhibiting the PI3K/AKT/mTOR pathway.

## Discussion

DLBCL is a highly heterogeneous disease in terms of morphology, genetics, molecular pathology, and clinical symptoms, with numerous factors influencing its prognosis. In recent years, as the incidence of DLBCL has risen, so have its recurrence and mortality rates. Despite the development of new diagnostic techniques and targeted therapies, nearly half of newly diagnosed patients fail to achieve complete remission. The R-CHOP regimen, a first-line therapy, can slow disease progression but demonstrates limited effectiveness for recurrent and refractory DLBCL. Moreover, it is associated with adverse effects, including increased clotting risk, reduced fertility, and impacts on other conditions [27–30]. This underscores the urgent need to identify novel therapeutic targets and biomarkers to improve DLBCL diagnosis and treatment outcomes. Apolipoproteins, key components of plasma lipoproteins, bind to lipids and facilitate their transport to various tissues for metabolic use. They are involved in processes, such as atherosclerosis and cardiovascular and cerebrovascular disorders [31]. Apolipoproteins also influence cancer-related pathways, including PI3K/Akt, MAPK, and Wnt, playing crucial roles in carcinogenesis and tumor progression [11, 13]. APOC family—comprising APOC1, APOC2, APOC3, and APOC4—exists in very low-density lipoproteins, chylomicrons, and high-density lipoproteins [32]. Among these, APOC1 is notably overexpressed in various malignancies, often correlating with poor patient outcomes [8, 33–37]. Research has shown that APOC1 increases vimentin expression and decreases E-cadherin levels, promoting EMT in breast cancer cells [38]. Suppressing APOC1 significantly reduces prostate cancer cell growth and colony formation [39], while its overexpression enhances glioblastoma cell proliferation, migration, and invasion [40]. In our study, bioinformatics analysis revealed that APOC1 is highly expressed in DLBCL, a finding further confirmed in clinical samples, where APOC1 levels were significantly higher in DLBCL samples compared to RHL samples. Ann Arbor staging and Kaplan–Meier survival analysis demonstrated that elevated APOC1 expression is strongly associated with advanced clinical stages and decreased five-year overall survival in DLBCL patients, suggesting it as a prognostic indicator. Additionally, reducing APOC1 expression inhibited malignant growth of DLBCL cells and significantly suppressed tumor growth in mice. These findings highlight APOC1 as a potential therapeutic target for DLBCL, offering both prognostic value and anti-tumor activity. Targeting APOC1 could thus represent a promising strategy to improve outcomes for DLBCL patients.

Cancer cells metastasize from the primary tumor to distant sites via endovascular circulation, during which HIF increases VEGFA release, driving tumor angiogenesis [41, 42]. VEGFA is a key regulator of pathological artery development, promoting endothelial cell mitosis, survival, and vascular permeability. It also enhances the mobilization of bone marrow-derived EPCs, which migrate to sites of ischemia or vascular injury, differentiate into mature endothelial cells, and facilitate neovascularization [43–45]. VEGFA promotes angiogenesis by binding to VEGFR-1 and VEGFR-2 and their co-receptors, neuropilin-1 and -2 (NRP-1 and NRP-2) [46]. HUVECs are widely used as models to study vascular endothelial cell behavior and effectively replicate tumor angiogenesis processes [47–49]. In our study, HUVECs were treated with conditioned media from SU-DHL-4 and SU-DHL-8 cells following APOC1 knockdown. This treatment resulted in a reduction in both neovascularized tubule formation and VEGFA protein production. Notably, APOC1 is known to regulate macrophage M2 polarization, and polarized macrophages secrete various pro-angiogenic factors, including VEGFA, to promote angiogenesis [8, 50]. The inhibition of angiogenesis observed in HUVECs following APOC1 knockdown is likely due to this regulatory mechanism. *In vivo* experiments using nude mice demonstrated that APOC1 knockdown significantly reduced the expression of endothelial differentiation markers CD31 and VEGFA in tumor tissues. These findings suggest that APOC1 facilitates the malignant metastatic spread of DLBCL by promoting tumor angiogenesis, ensuring robust intra-tumor circulation and cancer cell viability. This aligns with evidence that high APOC1 expression enhances the secretion of cytokines, such as EGF, PDGF, VEGFA, IL-1, IL-8, TNF-α, MMP-9, MMP-2, and NO, which collectively drive tumor cell proliferation, invasion, and angiogenesis [51]. Thus, APOC1 knockdown shows promise in disrupting DLBCL progression by impairing angiogenesis.

**Figure 7.**
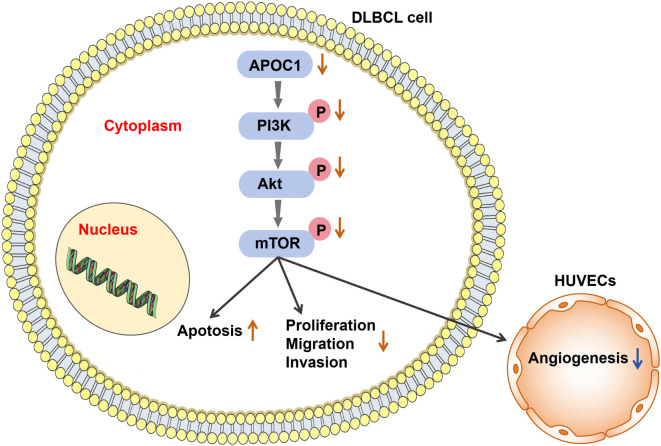
**Diagram of the mechanism.** Knockdown of APOC1 led in inhibition of the PI3K/AKT/mTOR pathway, which promoted apoptosis and decreased the malignant biological characteristics (proliferation, migration, and invasion) of DLBCL cells, as well as inhibited DLBCL cell angiogenesis. APOC1: Apolipoprotein C1; DBLCL: Diffuse large B-cell lymphoma; HUVEC: Human umbilical vein endothelial cell.

The PI3K/AKT/mTOR signaling pathway, which regulates cellular metabolism and cytoskeletal rearrangement, plays a critical role in human malignancies [27]. Among the key molecules implicated is PI3K, an intracellular phosphatidylinositol kinase associated with oncogenic proteins, such as Src and RAS. PI3K contributes to the development of nearly 50% of malignant tumors [52]. Upon activation, PI3K phosphorylates phosphatidylinositol 4,5-bisphosphate to phosphatidylinositol 3,4,5-trisphosphate, which facilitates the recruitment of AKT proteins to the cell membrane for phosphorylation. Activated AKT translocates to the cell membrane, where it stimulates protein synthesis, further promoting tumor progression. mTOR, a key downstream target of AKT, serves as a central regulator of multiple tumor cell signaling pathways [53, 54]. Our research demonstrated that knockdown of APOC1 significantly reduced p-PI3K, p-AKT, and p-mTOR levels in SU-DHL-4 and SU-DHL-8 cells, as observed through Western blot and immunofluorescence assays. These findings suggest that APOC1 knockdown disrupts the PI3K/AKT/mTOR signaling pathway. Additionally, an *in vivo* study showed that APOC1 knockdown in nude mice reduced the expression of Ki67, CD31, and VEGFA in subcutaneous tumor tissues. This was accompanied by a corresponding decrease in PI3K/AKT/mTOR pathway activity, providing further evidence that APOC1 influences this pathway. Previous studies have also highlighted the involvement of the PI3K/AKT/mTOR pathway in tumor angiogenesis. Activation of this pathway increases VEGF secretion through both HIF-1-dependent and independent mechanisms. It also regulates angiogenesis by modulating NO and angiopoietin expression [55]. Building on these findings, our results underscore the potential of targeting APOC1 to inhibit tumor angiogenesis by suppressing the PI3K/AKT/mTOR pathway. This strategy could effectively interfere with multiple malignant processes in DLBCL, paving the way for novel, personalized cancer therapies. Our experimental data further elucidated the role of APOC1 in DLBCL cells. Specifically, APOC1 knockdown in the cytoplasm of DLBCL cells led to reduced phosphorylation of PI3K/AKT/mTOR pathway proteins, thereby inhibiting angiogenesis, promoting apoptosis, and suppressing cell proliferation, migration, and invasion. The proposed mechanism of action is illustrated in Figure 7.

## Conclusion

Taken together, these findings suggest that knocking down APOC1 can promote apoptosis in DLBCL cells and reduce their proliferation, migration, invasion, angiogenesis, and tumor growth via the PI3K/AKT/mTOR signaling pathway. APOC1 may serve as a crucial diagnostic and therapeutic target for DLBCL, supporting its prevention, early diagnosis, precise treatment, and improved prognosis. Currently, there are no APOC1-related drug formulations available. In the future, increased investment in research and development will be necessary to advance the creation of APOC1-targeted therapies, evaluate their safety through clinical trials and toxicity tests, and determine the optimal dosage for administration to facilitate the clinical translation of these research outcomes. Finally, the journey toward a cure for DLBCL remains challenging and complex. Future research should focus on combining APOC1-targeted therapies with other treatments, including targeted, immunologic, and conventional medications. Investigating the effects of multidrug combinations will also be critical to maximizing the benefits for DLBCL patients.

## Data Availability

The data that support the findings of this study are available from the corresponding author, upon reasonable request.
